# Empirically identified networks of healthcare providers for adults with mental illness

**DOI:** 10.1186/s12913-021-06798-2

**Published:** 2021-08-06

**Authors:** Joshua Breslau, Beth Dana, Harold Pincus, Marcela Horvitz-Lennon, Luke Matthews

**Affiliations:** 1 RAND Corporation, 4570 Fifth Avenue, Pittsburgh, PA 15213 USA; 2 RAND Corporation, 20 Park Plaza #920, Boston, MA 02116 USA; 3 Department of Psychiatry, Columbia University, 1051 Riverside Drive, New York, NY 10032 USA

**Keywords:** Mental illness, Network analysis, Provider networks, Continuity of care

## Abstract

**Background:**

Policies target networks of providers who treat people with mental illnesses, but little is known about the empirical structures of these networks and related variation in patient care. The goal of this paper is to describe networks of providers who treat adults with mental illness in a multi-payer database based medical claims data in a U.S. state.

**Methods:**

Provider networks were identified and characterized using paid inpatient, outpatient and pharmacy claims related to care for people with a mental health diagnosis from an all-payer claims dataset that covers both public and private payers.

**Results:**

Three nested levels of network structures were identified: an overall network, which included 21% of providers (*N* = 8256) and 97% of patients (*N* = 476,802), five communities and 24 sub-communities. Sub-communities were characterized by size, provider composition, continuity-of-care (CoC), and network structure measures including mean number of connections per provider (degree) and average number of connections who were connected to each other (transitivity). Sub-community size was positively associated with number of connections (r = .37) and the proportion of psychiatrists (r = .41) and uncorrelated with network transitivity (r = −.02) and continuity of care (r = .00). Network transitivity was not associated with CoC after adjustment for provider type, number of patients, and average connection CoC (*p* = .85).

**Conclusions:**

These exploratory analyses suggest that network analysis can provide information about the networks of providers that treat people with mental illness that is not captured in traditional measures and may be useful in designing, implementing, and studying interventions to improve systems of care. Though initial results are promising, additional empirical work is needed to develop network-based measures and tools for policymakers.

**Supplementary Information:**

The online version contains supplementary material available at 10.1186/s12913-021-06798-2.

## Introduction

Adults with mental illnesses frequently receive care from multiple medical providers due to the complexity of their behavioral health needs and high prevalence of physical health comorbidities [1]. Recognizing this fact, policy efforts that aim to improve the care for this vulnerable population focus, explicitly or implicitly, on shaping the network of relationships among these providers. These efforts are particularly important in the United States, where there is a wide diversity of providers of both mental health and physical health care that are financed through a complex system of private and public insurances [2]. For instance, the behavioral health home model, in which primary care services are provided within specialty behavioral health clinics, envisions a care network organized into a hub, the health home clinic, with spokes, the ancillary and specialty services that are provided to individual patients with central coordination [3].

Other policy efforts also have implications for network structure that may be underappreciated. Care coordination services [4] provide patients with a new network connection, the care coordinator, that aims to serve as a hub between multiple providers, maintaining consistency and adequate coverage of the patient’s needs. Improving information sharing between providers through shared electronic health records [5] may shape networks by selectively facilitating communications among providers with access to the shared resource. Despite policy interest in organizational integration, defined as “the availability and functionality of linking structures that enable and sustain clinical integration” [1], little is known about existing provider networks and how they differ from one another.

Methods from social network analysis can be a useful approach to understanding networks of health care providers [6]. These methods identify networks empirically rather than administratively; networks are defined by the sharing of patients among providers rather than by formal administrative units, such as employment by the same health care system or facility [7–9]. Social network methods have been used to study provider networks with respect to opioid prescribing [10] and treatment of prostate cancer [11], but have yet to be applied more generally to the complex care needs of adults with mental illnesses [7]. Given the complexity of care for this population, which includes specialty and non-specialty mental health care in addition to general medical care, network methods could prove particularly valuable. For instance, network methods could be useful to state Medicaid policymakers in assessing the impacts of financial integration, such as behavioral health carve-ins and accountable care organizations, on clinical integration of care. Describing these networks is an important first step towards developing reliable tools for use in policy design, research, and system improvement.

In this paper, we apply social network models to an all-payer claims dataset on care provided to patients with mental illness in a single U.S. state to begin building the knowledge base for empirical investigations. Provider networks are identified using information on the number of patients that providers have in common; the more patients two providers have in common, the stronger is their link in the provider network. Data from all-payers is critical to this endeavor so that complete networks of providers can be identified. We examine variation across the identified networks in their structural characteristics that may influence integration between mental and physical health care and continuity of care, a measure of the extent to which a patient’s care is consistently provided by the same provider [12, 13]. Understanding how provider networks vary from one another with respect to these characteristics is a vital step towards developing an empirical approach linking network structures with healthcare processes and outcomes such as quality and cost, and whether and how care integration can be influenced by health policy.

## Methods

### Data source

Insurance claims data for calendar years 2016 and 2017 were obtained from the Colorado All-Payer Claims Database (CO-APCD). Colorado health care payers must submit data on every covered plan member who is a Colorado resident. A resident is any eligible subscriber whose residence is in Colorado plus their covered dependents, or students enrolled in a student plan for a Colorado college or university regardless of their official residence.

The CO-APCD includes paid inpatient, outpatient, and pharmacy claims from commercial insurers and the two largest public insurances, Medicare, which covers all Americans age 65 and over, and Medicaid, which covers low-income Americans. It does not include the uninsured or self-insured or those covered by self-insured employer plans, the Veterans Health Administration, TRICARE (the civilian component of the U.S. Military Health System), or the Indian Health Services. The dataset includes information on member eligibility, provider specialty, and geographic location. Members and providers can be uniquely identified across all payers, claims, and other sources of information.

### Study cohort

Our analytic dataset captured all claims for 717,200 patients who had at least one claim with a behavioral health diagnosis, defined by an ICD-10 code for a “mental, behavioral and neurodevelopmental disorders” (F01-F99 )[14]. The total population of Colorado was about 5.5 million in 2016. Exclusion of patients who did not meet criteria for age, diagnosis, receipt of care from provider types of interest in either 2016 or 2017 reduced the number of patients to 490,509. Age criteria required that patients be at least 18 years old at the beginning and less than 65 years at the end of the year. Diagnosis criteria excluded individuals whose only behavioral health diagnoses were nicotine dependence, neonatal abstinence syndrome or fetal alcohol syndrome. Provider type criteria required that a patient have at least one claim from one of the four provider types described below.

### Variable definitions

#### Provider type

We used the National Uniform Claim Committee (NUCC) Health Care Provider Taxonomy Code Set Version 19.1(National Uniform Claim [15]) to classify providers into four mutually exclusive specialty groups: psychiatrists, psychologists, behavioral health specialists, and primary care providers. We defined psychiatry to include holders of an MD who deal specifically with mental illness and psychology as PhD providers of care for mental illness. Behavioral health specialists include non-PhD providers for mental health interventions, such as social workers and counselors. Primary care providers included MD holding physicians of internal medicine, general practitioners, and nurse practitioners.

#### Bice-Boxerman continuity of care index

The Bice-Boxerman Continuity of Care Index (CoC) is one of a group of measures that are highly correlated with one another that are used to measure the degree to which a patient’s healthcare visits for a specific time period are with a single provider or practice group [16, 17]. CoC ranges from 0 to 1, with higher scores indicating greater care continuity. It is measured at the level of the patient, with each patient characterized with a CoC value. Specifically, we calculated CoC by implementing the following equation:

$$ {\displaystyle \begin{array}{l}\frac{\left({\sum}_i^{\mathrm{p}}={1}^{n\frac{2}{i}}\right)-n}{n\left(n-1\right)}\\ {}\end{array}} $$

Where *p* = total number of providers encountered by the patient.

*n* = total number of visits with any provider by the patient.

*n*_*i*_ = number of visits with provider *i* by the patient.

To construct the CoC, we identified outpatient visits using the National Committee for Quality Assurance (NCQA) outpatient code value set. This code set consists of current procedural terminology (CPT) codes in the Evaluation and Management Services code range (99201–99,499), Healthcare Common Procedure Coding System (HCPCS) codes, and uniform billing revenue codes (services at clinics and urgent care centers). The CoC was calculated separately for the 2016 and 2017 samples to standardize the time period. Only one outpatient visit per day for each patient-provider combination was counted. Following common practice, CoC was calculated for patients who had four or more visits in a year to avoid high variability stemming from patients with low utilization [18].

#### Demographic characteristics

Patient demographics included age, gender, and insurance coverage. Insurance coverage was based on all claims and classified as: Medicaid only, Medicare only, dual Medicaid/ Medicare, commercial only, and combination of commercial plus Medicaid or Medicare. Since more than 40% of members were missing race/ethnicity, this variable was not used.

#### Behavioral health diagnosis

Diagnoses were defined using the Clinical Classifications Software Refined (CSSR), Version 2019. 1[19]. The CCSR classifies more than 70,000 ICD-10 diagnosis codes into 21 clinically meaningful body systems, including the mental, behavioral and neurodevelopmental disorders (MBD) system. The MBD system identifies 34 disorder types, which we grouped hierarchically into six mutually exclusive categories in the following order: schizophrenia or other non-affective psychotic disorder, bipolar disorders, depression or other (non-bipolar) mood disorder, anxiety or stress-related disorder, other mental disorders, and substance use disorders (SUD). Supplemental Table 1 shows the definition of these categories from the CCSR codes. For each yearly analysis sample, we classified patients into the highest applicable category in the disorder hierarchy using all claims (individual provider and facility) with service start dates in the year and all ICD-10 diagnoses (principal and secondary) from these claims.

#### Network analysis

Connections between individual providers were defined by counting the number of patients they shared in common [8, 9]; based on prior methodological research, a threshold of three shared patients was required to consider two providers to be connected to each other [20]. We then extracted the largest connected component of the network after applying the 3 or more shared patient threshold. The largest connected component of a network is the largest unit in which one can move from any node (physicians) to any other node via network connections (3 or more shared patients).

We used the resulting list of provider-to-provider connections from the largest connected component for community inference, which we conducted with the algorithm of Clauset et al. implemented with the cluster_fast_greedy function in the igraph package for R [21]. Community detection is similar to nonhierarchical cluster analysis (e.g. k-means clustering), but where the input data are the presence/absence of connections between nodes rather than the differences among nodes in measured features (the typical input for clustering). As with nonhierarchical cluster analysis, community detection both determined the number of communities and the assignment of each node uniquely to a single community. The Clauset et al. algorithm, and most community detection algorithms, chooses the number of communities so as to maximize a network measure called *modularity*, which expresses the proportion of connections that exist within communities rather than between them. Using this algorithm, we first inferred the modularity maximizing *communities* for the overall network. We then constructed separate networks from each of these communities, and ran the algorithm again on each of them separately, in order to construct *sub-communities* that were nested within the first level. At each step, communities and sub-communities with fewer than 10 providers were excluded from further analysis.

Networks were characterized using metrics that have a long history within social network analysis. Network degree is the total number of other providers to whom a provider is connected. A provider who shares patients with a larger group of other providers has higher degree than a provider who shares patients with a smaller group of other providers, even if they see the same number of patients. A network with higher average degree has more connections per provider than a network with lower average degree. Because it was pertinent to our hypotheses about care integration for patients living with SMI, we also examined the number of connections (i.e. degree) from psychiatrists to primary care providers.

Transitivity is the proportion of a provider’s connections who are connected to each other. A network with higher transitivity may enable care coordination more efficiently, due to the high frequency of mutual connections. Lower transitivity indicates a more dispersed network. Transitivity was calculated with igraph.

Associations between network characteristics and network size, i.e. the number of providers in a network, were assessed using correlation coefficients. Both Pearson and Spearman correlation coefficients were calculated. We ran all analyses in R version 3.5.2 on a 64 bit redhat build for a linux server.

To explore the relationship between network measures and a traditional measure of the organization of care across multiple providers, we examined the relationship between network transitivity and CoC at the provider level. To account for clustering of patients within providers, we estimated the following multilevel model:

1 $$ CoC\sim I+{\beta}_1T+{\beta}_3P+{\beta}_4S+{\beta}_5{A}_{CoC}+u+e $$

Where, *CoC* is the continuity of care of each provider averaged across all patients with whom they had healthcare interactions as evidenced by paid claims, *I* is the intercept, *T* is the transitivity of each provider, *P* is the number of patients with whom a provider had healthcare interactions, *S* is the provider specialty, *A*_*CoC*_ is the average CoC of each individual’s direct social ties, and *u* is a random intercept for the network community assignment of each provider, and *e* is the residual error. Models were estimated with (Model 2) and without (Model 1) A_CoC_ to examine the impact of local network conditions. The purpose of this equation was to assess the level of association between CoC and transitivity after controlling for basic volume and specialty characteristics of the providers. We wanted to understand whether these measures are really correlated after these controls, and whether transitivity reflects only the individual physician’s own average CoC across their patients or if it is additionally related to the average CoC of the physicians with whom that physician shared patients. It seemed plausible that transitivity might be reflective of both because it is by definition a measure of local network clustering that surrounds each individual.

## Results

The initial model considered physicians to be connected if they shared 3 or more patients in common. After applying this threshold, we retained only the physicians who were in the largest connected component of the network, meaning the largest component in which one can move from any physician to any other via connections of shared patients. This largest component included 21% of providers (*N* = 8256) but retained 97% of patients (*N* = 476,802). This indicated that the vast majority of SMI patients in Colorado flow among the set of ~ 8000 physicians we identified through this analysis.

### Network composition

The patients included in the provider network are described in Table 1. The largest diagnostic category is depression or other mood disorder. About 6% of the sample had a diagnosis of schizophrenia or other psychotic disorder and 8% had a diagnosis of a bipolar disorder. With respect to insurance type, the two largest groups of patients, those with Medicaid and those with commercial insurance, together accounted for about 80% of the sample. The majority of the remaining patients had either Medicare or dual Medicaid-Medicare coverage.

**Table 1:** Sample Characteristics, Colorado All-Payer Claims Data on Patients with a Mental Health Diagnosis, 2016–2017 (*N* = 476,802)

Characteristic	N	%
Sex		
Female	295,618	62.0
Male	180,563	37.9
Missing	621	0.1
Age in 2016		
17 to 29^1^	117,782	24.7
30 to 40	118,902	24.9
41 to 51	110,361	23.2
52 to 63	129,757	27.2
Diagnosis		
Schizophrenia or Other Psychotic Disorder	29,887	6.3
Bipolar Disorder	98,984	8.2
Depression or Other Mood Disorder	205,378	43.1
Anxiety or Stress-Related Disorder	146,790	30.8
Other Mental Disorder	37,031	7.8
Substance Use Disorder	18,732	3.9
Insurance Coverage		
Medicaid only	161,843	33.9
Medicare only	21,698	4.6
Dual Medicaid/Medicare	42,514	8.9
Commercial Only	223,103	46.8
Commercial + Public	27,644	5.8

Diagnoses are defined hierarchically as described in the text. Insurance coverage is categorized for the 2-year period and includes simultaneous coverage as well as sequential change in coverage

^1^ Includes people who turned 18 during 2016 whose data during 2017 was included in the analysis

The provider network was comprised of 3833 primary care providers, 823 psychiatrists, 498 psychologists, and 3102 behavioral health specialists. On average, providers were connected to 60 other providers in the network (i.e. mean degree), with about half, 32, being connections with primary care providers (Table 2). The median number of connections, 26, was considerably below the mean, indicating that there is a relatively small group of providers with very high numbers of connections (Supplemental Fig. 1 shows the distribution of number of network connections across providers). Providers saw on average 179 patients in the network. Network transitivity, the proportion of a provider’s connections who are connected to each other, averaged 60%.

**Table 2:** Characteristics of providers in the mental health network in Colorado from 2016 to 2017

Variable	Minimum	Median	Mean	Maximum
Number of connections (network Degree)	1	26	59.54	1011
Proportion connections connected (Transitivity)	0	0.58	0.60	1
Number of connections to PCPs	0	12	31.82	681
Number of connections to PCPs in same network community	0	11	28.73	506
Number of patients	3	91	178.98	3356
Number of patients had claims with a PCP	0	41	113.07	1684
Number of Patients had claims with a PCP in same network community	0	38	108.40	1682

*N* = 8256 for all network measures. N for average Continuity of Care = 8252. PCP=Primary Care Provider

Within the overall network we identified 5 large provider communities. Network communities are selected so as to optimize the assortment of connections to be between providers within the same community rather than between providers in different communities. “Modularity” is a statistical measure of the extent to which the clusters outputted by the community detection algorithm accomplish this assortment. Ranging from 0 to 1, modularity is not merely the proportion of ties within communities; rather, it is a chance-corrected proportion after assuming a random model of tie placement, on which a community detection algorithm would still find statistical structure, but spurious structure. For more details, readers are recommended to Clauset et al. [21].

**Figure 1:**
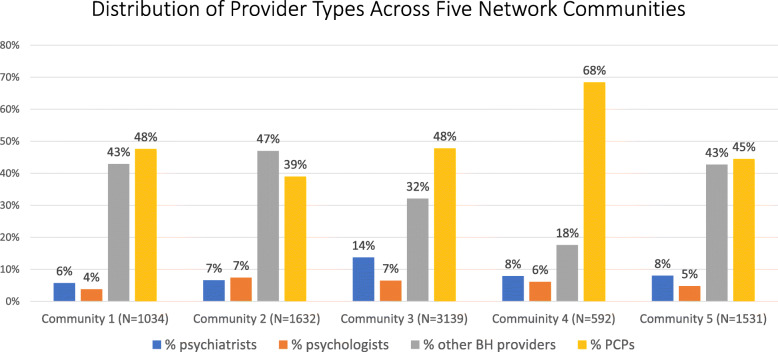
**Fig. 1** The five network communities range in size from 592 to 3139 providers. Primary care providers and other behavioral health providers are the most common provider types in all communities

At the level of the first 5 communities into which we split the total network, the modularity was 0.69. Thus, there was a clear preponderance of non-chance placement of shared patient connections within the 5 communities rather than between them. Communities ranged in size from 592 providers to 3139 providers (Fig. 1). The network communities tended to be comprised of large proportions of PCPs and Other BH providers, with relatively small proportions of psychiatrists and psychologists. The largest network community had the highest proportion of psychiatrists.

The five network communities were further divided into mutually exclusive sub-communities (Table 3). The modularity score resulting from these splits ranged from 0.28 to 0.69. Twenty-Four sub-communities with 10 or more providers were identified, with between 4 and 7 sub-communities per community. These 24 sub-communities included 7925 providers, 96% of all providers in the overall network. The sub-communities ranged in size within and between communities; the smallest sub-community had only 14 providers while the largest had 1320 providers. The sub-communities also differed within and between communities with respect to measures of network structure. The average number of providers to which each provider is connected ranged from 5 to 106. The average number of PCPs to which each provider is connected ranged from 2 to 79. The proportion of connections to PCPs within rather than outside of the sub-community was high in all communities; it ranged from 67 to 100%. Network transitivity ranged from 39 to 77%. In most of the network sub-communities the average continuity of care ranged from .25 to .33, but the sub-communities of community 4 had lower continuity of care, ranging from .16 to .25.

**Table 3:** Characteristics of Network Sub-Communities across 5 Communities

Network Community	Number of sub-communities	Sub-Community Size	Mean Number of Connections	Mean Connections to PCPs	% PCP Connections within sub-community	Mean Proportion of Connections Connected to Each Other	Average Continuity of Care	Modularity calculated across sub-communities
1	4	87 to 429	18 to 71	11 to 31	91 to 100	.62 to .70	.26 to .31	0.33
2	5	14 to 819	10 to 92	6 to 27	75 to 100	.51 to .77	.25 to .35	0.50
3	7	16 to 1320	8 to 56	2 to 37	67 to 100	.42 to .65	.25 to .32	0.43
4	4	15 to 281	5 to 106	4 to 79	89 to 100	.39 to .58	.16 to .25	0.28
5	4	83 to 683	30 to 85	14 to 31	90 to 97	.61 to .69	.28 to .39	0.69

PCP=Primary Care Provider

To assess the extent to which measures of network structure are independent of the size of network communities (number of providers), we calculated correlations between network characteristics and total number of providers across the 24 network sub-communities (Table 4). Community size is not mathematically inherently linked to network structural features other than the chance-corrected tendency of social connections to be clustered within, rather than between, communities [21]. Pearson correlation coefficients indicate weak (i.e. between 0.3 and 0.5) linear correlations between the number of providers in a community with mean number of connections but very weakly correlated or uncorrelated (under 0.3) with all other network structure measures. Spearman correlations, which do not assume linear relationships, are higher for correlations between number of provider and number of connections, but similar to Pearson correlations for other measures. Notably, the Pearson correlation between number of providers in a community and network transitivity is − 0.02. Number of providers is weakly correlated with the proportion of psychiatrists in a network, and very weakly correlated or uncorrelated with other network composition measures.

**Table 4:** Correlations of number of providers per community and community-level network characteristics (*N* = 24 network communities)

Network Characteristic	Pearson Correlation Coefficient	Spearman Correlation Coefficient
Network Structure		
Mean # connections	0.37	0.66
Mean # connections to PCPs	0.24	0.65
% Connections within network	0.12	−0.04
Network Transitivity	−0.02	0.08
Average Continuity of Care	0.00	0.03
Network Composition		
Psychiatrists	0.42	0.30
PCPs	−0.02	−.011
Psychologists	−0.18	0.09
Other BH Provider	0.03	0.09

PCP=Primary Care Provider, BH=Behavioral Health

Among providers, the unadjusted Pearson correlation between network transitivity and CoC is 0.08 (Spearman rho = 0.7). In our model 1 adjusting only for provider type and number of patients, transitivity is significantly negatively associated with CoC indicating that higher transitivity is associated with lower continuity of care; a one SD increase in transitivity associated with a decrease of 32% of an SD in CoC (*p* = .006) (Table 5). However, after adjusting for the average CoC of each provider’s connections (Model 2), this association is reduced in magnitude and no longer statistically significant.

**Table 5:** Adjusted relationship between transitivity and continuity of care

	Model 1		Model 2	
Variable	Slope	p-value^1^	Slope	p-value^1^
Intercept	0.25	< 0.001	−0.032	< 0.001
Transitivity	−0.011	0.006	0.00062	0.85
Number Patients (P)	0.0000060	0.26	−0.00000022	0.96
Provider Type				
BH Specialist	0.061	< 0.001	0.021	< 0.001
Psychiatrist	0.027	< 0.001	0.010	< 0.001
Psychologist	0.034	< 0.001	0.017	< 0.001
PCP	Reference		Reference	
Alter CoC			1.1	< 0.001

^1^P values are for Wald statistics with 1 degree of freedom

BH=Behavioral Health, PCP=Primary Care Provider

## Discussion

Mental health policymakers and researchers have long recognized the importance of provider networks, explicitly and implicitly, in the design of interventions, treatment models, and evaluation studies. However, methods for understanding the structure and functioning of provider networks and their effects on care that patients receive are generally limited to patient and provider or clinic level analyses. This paper is the first attempt to use network modeling methods to formally describe provider networks in this complex area of the health care system. Our goal was to identify and characterize provider networks and assess whether they vary in potentially modifiable ways that might impact the care that patients receive. Our focus on a single U.S. state allowed us to study networks operating within a relatively homogeneous administrative environment. Many aspects of health care systems in the United States are regulated or administered by state agencies, including administration of Medicaid, which finances care for low-income patients. The results provide a strong indication that there is variation across networks defined at multiple levels that is not captured by traditional measures of care. The findings suggest some hypotheses and methods for testing them that can advance our empirical knowledge about integration of care.

The initial step was to identify and describe the overall network of providers caring for people with mental illness in the state. The model confirms and expands the knowledge base regarding treatment for people with mental illnesses. The overall network of providers who treat mental illness is comprised of a small portion of the providers in the state (21%), and this network provides care to nearly all the patients (97%) treated for mental illness in the state. The remaining providers treat very small numbers of patients with mental illnesses and do so in relative isolation from colleagues. Providers who see patients with mental illnesses are connected through those patients to a large number of other providers; the median provider shares patients with mental illness with 26 other providers. Of these about half are connections to primary care providers, indicative of the large portion of the care received by people with mental illness that is for physical health conditions [22, 23]. The large number of connections between providers indicates the challenge of integrating care across the system.

Connections between behavioral health and non-behavioral health providers are a particular concern for policymakers because of high rates of chronic physical health conditions in this population and the challenges of integrating care across sectors of the health care system. We find that the large majority of connections to PCPs were to PCPs within the network, as opposed to outside of the network. This finding suggests an interesting question for future research; is the proportion of connections to PCPs that are within the network associated with better quality of care for physical health conditions resulting from improved coordination. There may be a benefit to having primary care providers linked through provider networks to other providers who also see patients with mental health diagnoses.

Within this overall network, which covers the entire state, we identified two additional levels of structure, which we label communities and sub-communities. The community level comprises large groups of providers; it is unlikely that providers would be directly affected by changes that target this level of the network. The communities are similar with respect to the distribution of provider types, but there are some variations that could have implications for care. For instance, while the proportion of providers that are psychiatrists is always relatively low, this proportion varies more than 2-fold across the communities.

The next level of analysis identified what we call network sub-communities, which are of a size that could be reasonably targeted for intervention by state or local policy officials. The sub-communities are defined to be mutually exclusive and nested within the previously identified network communities. At this level the networks are heterogeneous with respect to size and structure, and these variations suggest that there may be modifiable aspects of the networks that could be related to quality of care. Some of the structural features of the networks are associated in a predictable way with the size of the network. For instance, network size is positively associated with the average number of connections among the providers in the network. There are policies that could increase or decrease network size. For instance, strengthening incentives to provide care in defined networks could divide the overall network into a larger number of smaller sub-communities. We do not yet have empirical evidence supporting this hypothesized impact of network policies.

There are also structural features of the networks that are independent of network size. In particular, transitivity, i.e. the proportion of a provider’s network connections who are connected to each other, is not correlated with network size. Transitivity can be thought of as a novel indicator of provider integration. Where transitivity is high, the network is highly interconnected by the sharing of patients; where transitivity is low, connections are relatively sparse. Empirical work is needed to assess the relationship between transitivity and other aspects of integrated care and the impact that policies might have on transitivity. It is interesting to note that different policies which aim to improve care integration might have different effects on network transitivity. Some models, such as accountable care organizations [24], aim to create relatively dense networks of integrated providers, which might also have the effect of separating networks from each other. Other models, such as New York’s Health Home model [25], focus on connecting individual patients with multiple providers without consideration of how the providers are related to one another. Further analysis of network models could provide empirical evidence regarding the relative values of these alternative approaches.

To assess whether the network transitivity identifies a feature of provider networks that is not captured by traditional measures, we examined its relationship with continuity of care, a traditional measure that also focuses on how multiple providers contribute to patient care. The Bice-Boxerman CoC index, assesses the proportion of a patient’s visits that are to a single provider and is highly correlated with other commonly used measures of CoC [17]. Higher CoC is associated with fewer hospitalizations and emergency room visits [26] and lower health care costs [27]. Our finding of a weak inverse association between average CoC and transitivity, which was attenuated and non-significant when controlling for the CoC of providers direct connections, suggests that network transitivity is not duplicating measurement of CoC and may provide a useful new measure of system function. That is, transitivity appears to reflect something about the average CoC of each provider’s connected providers (network “alters”) above and beyond what is captured by their own CoC measure. Future research would be needed to determine whether transitivity (or alternatively measured as average *network alter* CoC), has a relationship to care quality or cost that is not predicted by each provider’s own average CoC across their patients.

### Limitations

Limitations of the current study should be considered. First, we examine only one state. For this preliminary analysis, focusing on one state simplifies our analysis, but it is likely that provider networks vary across states, particularly in the public sector. In Colorado, as in some other states, public mental health services are systematically organized by catchment areas, while some states, such as New York, have much more varied systems of care provided by a diverse group of provider organizations. States also vary in the extent to which public mental health services are centralized or decentralized with strong county level administration. These organizational features might influence the structure of provider networks and moderate their impact on quality of care. Application of these methods to a more diverse group of states will help illuminate this type of variation and could be used to tailor intervention to these different institutional contexts.

Second, the structural features of provider networks may depend on the level at which networks are identified and analyzed. In this analysis we identified an overall network within the state, network communities within this overall network and an additional lower level of network sub-communities. Additional levels of organization of the overall network could be identified, and, in some cases, might prove useful to local policymakers and researchers. Additional work may reveal valuable lessons regarding smaller, lower level network groupings than were identified in this study. Iteration between network analysis and surveys or smaller qualitative studies could help build knowledge in this area.

Third, our dataset excludes care from the VA system, the Indian Health Service and self-insured employers. These are gaps that could be filled in future studies. The inclusion of both Medicaid and Medicare in a single dataset, which covers a large portion of people with mental illness who qualify for disability, is a strength.

## Conclusion

The health care system can be characterized by de facto networks of providers that are shaped by the cumulative impacts of myriad health organizations of diverse size and structure, informal aspects of clinical practice, and help-seeking practices of patients. This preliminary investigation suggests that network analysis methods applied in other areas of health care offer some promise for improving our ability to understand, study, and modify the complex care that patients with mental illness receive. Networks of providers and their characteristics may prove to be particularly important for mental health services, given the early onset, high prevalence of physical and mental comorbidity and the longstanding challenges of care in a fragmented health care system. Patients with mental illness tend to interact with the health system over many years, making the influence of provider networks particularly consequential. Additional empirical investigation and methods development are needed to explore whether network characteristics are related to quality of care, whether network analysis methods can detect impacts of policy on the health care system, whether network characteristics mediate or moderate policy impacts on quality and outcomes of care.

## Supplementary Information

**Additional file 1 Supplemental Table 1**. Hierarchical Definition of Mental Disorder Categories from CCSR Categories.

**Additional file 2 Supplemental Fig. 1**. Histogram of number of network connections for each provider (a.k.a. physician “degree distribution”).

## Data Availability

Data from the Colorado All-Payer Claims Dataset were obtained from the Center for Improving Value in Healthcare. Under our data use agreement, we are unable to share the data outside of the research team without specific advance approval from the state of Colorado. Information on accessing these data are available online from: https://www.civhc.org/get-data/co-apcd-overview/.

## Abbreviations

BH: Behavioral Health
CO-APCD: Colorado All-Payer Claims Database
CoC: Continuity of Care Index
CPT: Current Procedural Terminology
CSSR: Clinical Classifications Software Refined
HCPCS: Healthcare Common Procedure Coding System
MBD: Mental, Behavioral and Neurodevelopmental Disorders
NCQA: National Committee for Quality Assurance
NUCC: National Uniform Claim Committee
PCP: Primary Care Provider
