# Supplementary material for: Empirically identified networks of healthcare providers for adults with mental illness
Source: BMC Health Serv Res. 2021 Aug 6;21:777. doi: 10.1186/s12913-021-06798-2 (PMC8349008; doi:10.1186/s12913-021-06798-2)
Supplement: Supplementary file 1 — Additional file 1 Supplemental Table 1. Hierarchical Definition of Mental Disorder Categories from CCSR Categories. [file 12913_2021_6798_MOESM1_ESM.docx]

Supplemental Table 1: Hierarchical Definition of Mental Disorder Categories from CCSR Categories

| **CCSR Disorder Category*** | **Mental Disorder Category** |
| --- | --- |
| Schizophrenia spectrum and other psychotic disorders (CCSR MBD001) | Schizophrenia or Other Psychotic Disorder |
| Bipolar and related disorders (CCSR MBD003) | Bipolar Disorder |
| Other specified and unspecified mood disorders (CCSR MBD004) | Depression or Other Mood Disorder |
| Depressive disorders (CCSR MBD002) |  |
| Anxiety and fear-related disorders (CCSR MBD005) | Anxiety or Stress-Related Disorder |
| Trauma- and stressor-related disorders (CCSR MBD007) |  |
| Obsessive-compulsive and related disorders (CCSR MBD006) |  |
| Disruptive, impulse-control and conduct disorders (CCSR MBD008) | Other Mental Disorder |
| Personality disorders (CCSR MBD009) |  |
| Feeding and eating disorders (CCSR MBD010) |  |
| Somatic disorders (CCSR MBD011) |  |
| Suicidal ideation/attempt/intentional self-harm (CCSR MBD012) |  |
| Suicide attempt/intentional self-harm; subsequent encounter (CCSR MBD027) |  |
| Miscellaneous mental and behavioral disorders/conditions (CCSR MBD013) |  |
| Mental and substance use disorders in remission (CCSR MBD026) |  |
| Mental and substance use disorders; sequela (CCSR MBD034) |  |
| Opioid-related disorders (CCSR MBD018) | Substance Use Disorder |
| Opioid-related disorders; subsequent encounter (CCSR MBD028) |  |
| Alcohol-related disorders (CCSR MBD017) |  |
| Cannabis-related disorders (CCSR MBD019) |  |
| Cannabis-related disorders; subsequent encounter (CCSR MBD030) |  |
| Sedative-related disorders (CCSR MBD020) |  |
| Stimulant-related disorders (CCSR MBD021) |  |
| Hallucinogen-related disorders (CCSR MBD022) |  |
| Inhalant-related disorders (CCSR MBD023) |  |
| Other specified substance-related disorders (CCSR MBD025) |  |
| Stimulant-related disorders; subsequent encounter (CCSR MBD029) |  |
| Hallucinogen-related disorders; subsequent encounter (CCSR MBD031) |  |
| Sedative-related disorders; subsequent encounter (CCSR MBD032) |  |
| Inhalant-related disorders; subsequent encounter (CCSR MBD033) |  |
