# Supplementary material for: Empirically identified networks of healthcare providers for adults with mental illness
Source: BMC Health Serv Res. 2021 Aug 6;21:777. doi: 10.1186/s12913-021-06798-2 (PMC8349008; doi:10.1186/s12913-021-06798-2)
Supplement: Supplementary file 2 — Additional file 2 Supplemental Fig. 1. Histogram of number of network connections for each provider (a.k.a. physician “degree distribution”). [file 12913_2021_6798_MOESM2_ESM.docx]

Supplemental Figure 1. Histogram of number of network connections for each provider (a.k.a. physician “degree distribution”)


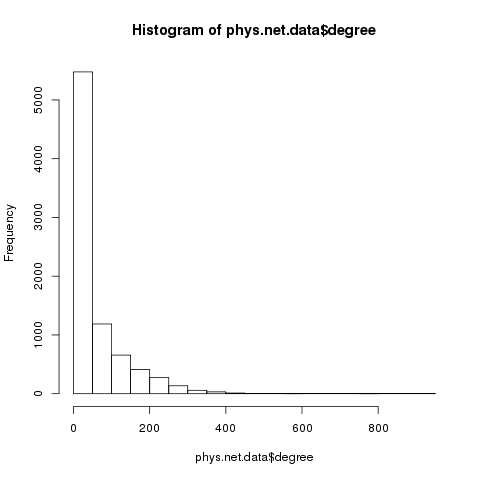

Provider number of connections (i.e. network degree) to other healthcare providers via ties of shared patients
